# Large-scale integration of small molecule-induced genome-wide transcriptional responses, Kinome-wide binding affinities and cell-growth inhibition profiles reveal global trends characterizing systems-level drug action

**DOI:** 10.3389/fgene.2014.00342

**Published:** 2014-09-30

**Authors:** Dušica Vidović, Amar Koleti, Stephan C. Schürer

**Affiliations:** ^1^Center for Computational Science, University of MiamiMiami, FL, USA; ^2^Department of Molecular and Cellular Pharmacology, University of MiamiMiami, FL, USA

**Keywords:** systems-biology, data integration, drug profiling, chemical similarity, kinome profiles, transcriptional signatures

## Abstract

The Library of Integrated Network-based Cellular Signatures (LINCS) project is a large-scale coordinated effort to build a comprehensive systems biology reference resource. The goals of the program include the generation of a very large multidimensional data matrix and informatics and computational tools to integrate, analyze, and make the data readily accessible. LINCS data include genome-wide transcriptional signatures, biochemical protein binding profiles, cellular phenotypic response profiles and various other datasets for a wide range of cell model systems and molecular and genetic perturbations. Here we present a partial survey of this data facilitated by data standards and in particular a robust compound standardization workflow; we integrated several types of LINCS signatures and analyzed the results with a focus on mechanism of action (MoA) and chemical compounds. We illustrate how kinase targets can be related to disease models and relevant drugs. We identified some fundamental trends that appear to link Kinome binding profiles and transcriptional signatures to chemical information and biochemical binding profiles to transcriptional responses independent of chemical similarity. To fill gaps in the datasets we developed and applied predictive models. The results can be interpreted at the systems level as demonstrated based on a large number of signaling pathways. We can identify clear global relationships, suggesting robustness of cellular responses to chemical perturbation. Overall, the results suggest that chemical similarity is a useful measure at the systems level, which would support phenotypic drug optimization efforts. With this study we demonstrate the potential of such integrated analysis approaches and suggest prioritizing further experiments to fill the gaps in the current data.

## Introduction

Modern molecular biomedical science relies to a great extent on understanding gene function, and significant progress was made in understanding the roles of numerous individual genes (Silverman and Loscalzo, 2012). However, the most critical unmet medical needs correspond to complex diseases caused by a combination of genetic and environmental factors, such as in cancer.

Many studies have demonstrated that cancer emerges from abnormal protein-protein, regulatory and metabolic interactions caused by concurrent structural and regulatory changes in multiple genes and pathways (Nagaraj and Reverter, 2011; Acencio et al., 2013). Further advances in the prevention, diagnosis and treatment of cancer require a more comprehensive knowledge of the molecular mechanisms that lead to the malignant state. Therefore, understanding cancer pathogenesis requires knowledge of not only the specific contributory genetic mutations but also the cellular framework in which they arise and function (Hong et al., 2008). Cancer cell lines and primary cancer cells have recently been established as powerful model systems to study cancer biology and the pharmacology of drug responses in cancer subtypes. To deconvolute, model, and understand drug sensitivity relies on systems-wide approaches to integrate large-scale biological responses in diseased and healthy cell states, involving various molecular entities such as drugs, proteins, genes, transcripts, cellular, and molecular processes, characteristics (e.g., genetic) of the cell model systems, etc. (Barretina et al., 2012; Heiser et al., 2012; Yang et al., 2013). Of particular interest for the development of novel drugs is their molecular mechanism of action (MoA). MoA describes biochemical interaction through which a drug modulates the corresponding target resulting in a phenotypic response (or pharmacological effect of the drug). Although there are studies linking drug pharmacology to transcriptional responses (Lamb et al., 2006), the connection to drug targets and the chemical structure of drugs is underexplored, partially because of a lack of large-scale profiling data. Such insights are of particular interest for the rational development of next-generation poly-pharmacology drugs (Hopkins, 2008). Here we present such a study based on data generated at the Library of Integrated Network-based Cellular Signatures (LINCS) project^1^.

It is one of the major goals of the LINCS project to generate an extensive reference set of cellular response signatures to representative small molecule and genetic perturbations that can facilitate the development of computational systems-level models of complex diseases and drug action. Common patterns from these data (signatures) include information about gene transcription, protein binding, cell proliferation, cell signaling and other cellular phenotypes with a particular focus on cancer. The LINCS data matrix extends into several dimensions including the model systems (cell lines, primary cells), the perturbations (such as small molecules), and the readout including the genome-wide transcriptional profiles, Kinome-wide binding profiles, and cell-viability and phenotypic profiles against a broad range of cell lines. These biological responses are currently generated, collected, and standardized to facilitate their integration. Data and tools generated in the LINCS consortium are available to the research community via the LINCS website (http://lincsproject.org). The integration of these data and their analysis relies on robust metadata standards developed at LINCS (Vempati et al., 2014). There are also a few recently published approaches that utilize specific LINCS data sets such as transcriptional profiles (Chen et al., 2013a,b) or kinase inhibition profiles (Shao et al., 2013).

Here we apply these standards and report their implementation with a focus on small molecules. We report several case studies involving multi-level integration of such diverse LINCS datasets. Based on large amounts of publically available kinase inhibition and binding data beyond LINCS, we built and applied computational models to fill gaps in the LINCS data matrix to enable much more comprehensive integrative data analyses. We demonstrate some global trends that link chemical features of small molecule perturbations, chemical biology, genomics and cell viability profiles illustrating the complexity and scope of LINCS data and how datasets can be mined. In several examples we show meaningful and biologically interpretable linkages among different signature types in the context of small molecule drugs and known signaling networks.

We hope that our survey and integrative analyses illustrates the wide scope and potential of the LINCS project and will motivate others to use LINCS generated data and knowledge to enhance their research on diverse biological and biomedical problems.

## Materials and methods

### LINCS assays and datasets

LINCS datasets cover a range of assays and technologies. Details about LINCS assays, data and tools are available at the LINCS project website (http://lincsproject.org/). For the analyses presented here we used three different types of LINCS data. All data used here can also be obtained via our LINCS Information FramEWork (LIFE) search system^2^.

#### Transcriptional response profiling data (L1000)

For the purposes of this study we selected two L1000 experiments (Peck et al., 2006) with fairly dissimilar cell lines, A549 (non-small cell lung carcinoma) with 1027 compounds tested, and VCAP (prostate carcinoma) with 741 compounds tested, in order to compare expression profiles among the same cell lines as well as between different ones. Although there is no simple measure of cell line similarity (LINCS is one of the first systematic efforts that contribute to the large-scale generation of cellular response signatures), for the purposes of this study we consider these cell lines in the basis of their origin from different organs. In total, here we investigate 1768 “is_ gold” signatures, corresponding to 1,729,104 data points (total number of Z-scores; perturbagens × transcribed genes measured × cell lines). All LINCS L1000 data and signatures are available at the Broad LINCS Cloud^3^. For more details on the L1000 data see Supplementary Material.

#### KINOME-wide binding profiles (KINOMEscan)

LINCS kinase biochemical profiles were generated at Harvard Medical School (HMS) using the DiscoveRx KINOMEscan technology^4^, which is a competition binding assay. A panel of 478 purified kinases was profiled against 78 small molecule compounds. However, the majority of LINCS compounds were not profiled in the KINOMEscan assay and we therefore generated predicted KINOME-wide inhibition/binding profiles based on classification models (described below).

#### Cell growth inhibition profiles

Cell growth inhibition datasets (assay developed at the Center for Molecular Therapeutics at Massachusetts General Hospital) (McDermott et al., 2007; Garnett et al., 2012) were retrieved from the LIFE database and the data were aggregated by averaging replicates. 39 small molecules were tested against 582 previously standardized cell lines at different concentrations (in the range from 0.004 to 15 μM) and one time point (72 h) and number of surviving cells counted. The measured cell viability values center around mean of 81% (corresponding to 19% growth inhibition) with a standard deviation of 31.68 across all concentrations.

### Small molecule chemical structure standardization, identification, and annotations

Compound information for small molecule perturbagens was received from the LINCS Data Production centers, HMS and Broad Institute. To identify unique and common compounds required a rigorous structure standardization pipeline that we implemented for the LINCS program. We used Pipeline Pilot 8.0 (Pipeline Pilot, 2011) components to generate the structures and remove addends and they were then subjected to the PubChem^5^ chemical structure standardization procedure using the Power User Gateway (PUG) service. In order to further identify PubChem CIDs we used additional service provided by PubChem PUG. The entire process was automated in a custom protocol using Pipeline Pilot. Using this process, a total of 5364 (as of October, 2013) unique LINCS compounds were obtained and LINCS small molecule (LSM) IDs assigned. More details on the procedure can be found in the Supplementary Material.

Additional information and annotations for the standardized structures were retrieved from PubChem but also from numerous external resources including DrugBank^6^, the NCBI^7^ MLP probe reports, the NCATS pharmaceutical collection (NPC), and the Protein Data Bank (PDB) (Berman et al., 2003). Compounds were annotated as approved drugs, kinase inhibitors, MLP probes, PDB ligands and, if information available, as kinase inhibitor of type I or type II (defined by the kinase ATP-binding site conformation in the ligand-bound form) (Dar and Shokat, 2011). All compound information can be queried, browsed and downloaded via the LIFE search system (http://life.ccs.miami.edu) and the LIFE project website^8^.

To characterize the diversity in chemical space of the tested LINCS compounds, we generated a histogram of their pairwise chemical similarities based on the Tanimoto metric using extended-connectivity fingerprints of length 4 (ECFP4) (Rogers and Hahn, 2010).

Based on unique LSM IDs we identified overlap of screened compounds among the different LINCS assays. While many compounds were tested in the L1000 gene-expression assay at the BROAD Institute, only few of those were tested in different assays at HMS.

### Small molecule kinase inhibitor models

We generated predicted kinase inhibition/binding profiles for all LINCS compounds to fill missing information of those compounds not (yet) tested in the HMS KINOMEscan assay. For that purpose we built Laplacian-corrected naïve Bayesian classification models using the procedure previously described (Schurer and Muskal, 2013); the models used here were rebuilt based on the new kinase inhibition data that doubled in the meantime illustrating rapid growth in published kinase inhibition data. Small molecule kinase activity data was extracted from the Q2 2013 release of the Kinase Knowledge Base (KKB, Eidogen-Sertanty)^9^. After standardization and aggregation based on unique kinases and compounds as previously described, the data amounted to more than 510,000 kinase structure data points with more than 270,000 actives (pIC50 > 6) and more than 590,000 total compounds covering the entire human Kinome. For each model, the number of total data points and actives was considered and only models for kinases with reasonable amount of data were built. For computational kinase profiling, we selected only models with the area under the receiver operating characteristic (ROC) curve greater than 0.9 and if they were based on at least 20 unique activity data points with 10 of them being considered active (pIC50 > 6). This selection resulted in 229 kinase models for which we could make confident predictions (for these 229 kinase models the additional information regarding their characteristics [target, number of data points, number of actives, ROC score, and enrichment factor for 1% for leave-one-out cross validation] can be found in Dataset 1 in the Supplementary Material). The model classifier outcome is a prediction of a compound being active (prediction value is true) or inactive (prediction value is false) for a given kinase. The outcome of performing all models against the LINCS compounds was converted into a 229-bit binary fingerprint for each compound.

### Kinase and small molecule kinase inhibitor annotations

To integrate KINOMEscan results and kinase models, we manually mapped them to Uniprot, standardized descriptions including mutations and posttranslational modification and we added external annotations such as protein name, symbols, IDs and alternate names, and also important details such as gatekeeper amino acid residues. We organized all kinase domains by an extended phylogenetic classification tree that we based largely on the Sugen kinase classification (Manning et al., 2002)^10^.

For LINCS standardized compounds a set of additional annotations were derived from the LINCS datasets. We defined active, selective, group selective and promiscuous kinase inhibitors based on the number and the group membership of kinases that are measured in the KINOMEscan assay. Compounds were considered active if they inhibited a kinase more than 90%. If a compound is active toward 5 or more kinases (belonging to different kinase groups) it was considered promiscuous. Compound was defined as selective kinase inhibitor if it is active toward only one kinase, or group selective if it was active only against kinases from the same kinase group. This data is available via the LIFE search system and the LIFE project website.

### Cell lines annotations

Numerous cancer cell lines and non-transformed primary cultures are used as disease model systems in the LINCS project. To facilitate integration and analysis of large-scale cell-based screening profiles generated at LINCS, cell lines were systematically annotated with controlled terms identifying associated organs and diseases (Vempati et al., 2014). Ongoing and future LINCS datasets are also being expanded toward primary tissues, iPS cells and their differentiated derivatives. Here we leverage disease annotations, which are available from the HMS LINCS website^11^, and can also be queried in the LIFE search system (http://life.ccs.miami.edu).

### Bioprofile- and chemical structure-based fingerprints and similarities

To facilitate comparative analysis of LINCS datasets, we defined several bioprofile fingerprints for tested compound. These bioprofile fingerprints were constructed based on categorical outcomes (active/inactive) in the different LINCS profiling assays. The Tanimoto metric was then used as a similarity measure of these profiles (similarities KinomeSim, KinomePredSim, and TranscriptSim for KINOMEscan, predicted kinase inhibition profile, and transcriptional expression profile, respectively). Advantages of this approach include simplicity (binary fingerprints) and computational efficiency (i.e., compute Tanimoto similarities). Chemical similarity of LINCS compounds (ChemSim) was determined based on topological fingerprints derived from the chemical structures also employing the Tanimoto metric. The definition of the fingerprints is provided in the Supplementary Material.

### Kinase enrichment in cell growth inhibition data

We integrated and analyzed the KINOMEscan data and cell growth inhibition assay data, which were retrieved from the LIFE database (http://life.ccs.miami.edu). KINOMEscan data consists of 78 small molecules tested against the panel of 478 kinases (including clinically relevant mutants, lipid, atypical, and pathogen kinases), corresponding to 382 unique kinase UniProt IDs. Cell growth inhibition data represents results of 39 small molecules tested in 582 cell lines (standardized as described above) at different concentrations (in the range from 0.004 μM to 15 μM) and one time point (72 h). Twenty one compounds were tested across the two described datasets and were used to integrate the data. For each kinase we calculated an enrichment score to reflect how much more likely it is to find activity in the cell growth inhibition assay among compounds that inhibit that particular kinase over the background probability of a compound inhibiting cell growth (the further details are provided in the Supplementary Material). Kinase enrichment scores were further used in the hierarchical clustering analysis performed by TIBCO Spotfire software (TIBCO Spotfire, 2013). Clustering was based on the single linkage method and the Euclidian distance was used as a distance measure.

### PI3K/AKT/mTOR pathway analysis

In order to demonstrate systems-level data integration, we considered kinases in the PI3K/AKT/mTOR signaling pathway (Laplante and Sabatini, 2012). We identified and downloaded 213 proteins (including cellular localization variation) from PI3K/AKT/mTOR pathway from Reactome (Joshi-Tope et al., 2005; Vastrik et al., 2007). By matching their genes to the standardized kinase genes symbols in the KKB, we identified 26 unique kinases. We then queried the aggregated KKB (the data that was also used for building the models) for those small molecules with a pIC50 value greater than 6 against any of these kinases and we identified 24,158 unique kinase inhibitors. Their (standardized) structures were compared to the LINCS compounds and we identified an overlap of 35 compounds. Based on the KKB activities, they inhibit 21 out of 26 PI3K/AKT/mTOR pathway kinases. For these 35 compounds that theoretically affect PI3K/AKT/mTOR pathway, we analyze their L1000 responses and the effect on the cell growth inhibition.

### Systematic pathway analysis

For the systematic pathway analysis our starting point was the curated pathway database of the National Cancer Institute (NCI)^12^. We retrieved the tab delimited file “NCI-Nature Curated Pathway–UniProt mapping” from their website (http://pid.nci.nih.gov/download.shtml). This file contains a total of 8420 records, which represent a combination of 2688 unique Uniprot IDs and 224 pathways (as of April 3, 2014).

In order to identify kinases, we grouped proteins by the pathways and compared their UniProt IDs to the kinase annotations in the KKB. For each pathway we further identified LINCS compounds that were predicted (by the kinase models, as described above) to be active for the kinases identified in the given pathway, and consequently active in that pathway. For such pathway-active compounds we compared their transcriptional similarities and computed *p*-values between TranscriptSim of pathway-active and pathway-inactive LINCS compounds in order to demonstrate that (predicted) pathway-active compounds lead to (statistically) significantly more similar transcriptional profiles than the pathway-inactive compounds.

### Student *T*-test calculations

All Student *t*-test calculations reported here were performed using the R Statistics^13^ component “R Two-Variables Tests” implemented in Pipeline Pilot 8.0.

## Results

### Characterization of LINCS small molecule perturbagens

**Figure 1:**
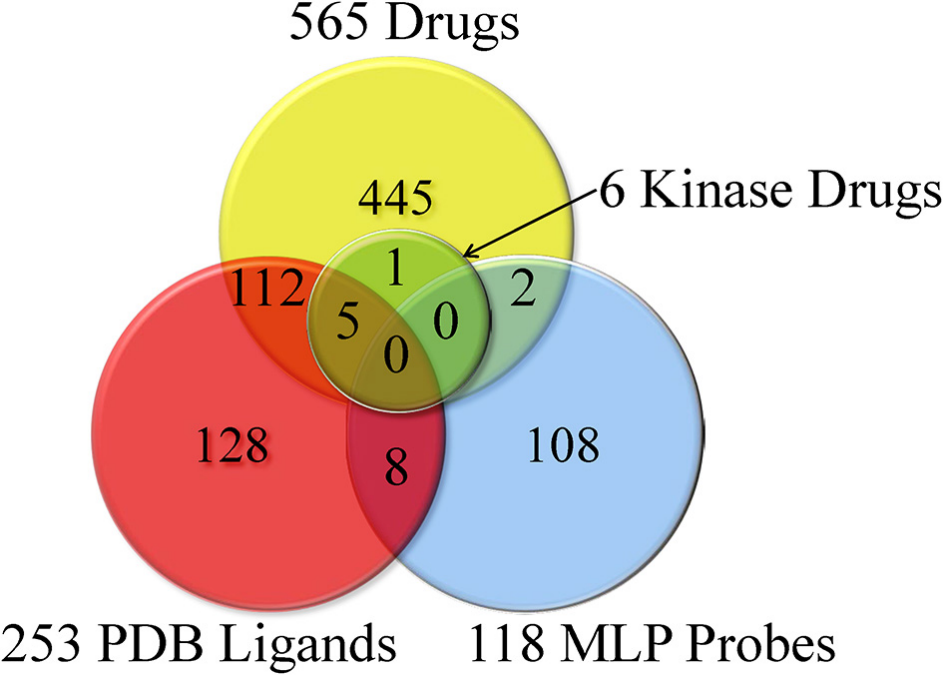
**Identified known PDB ligands, MLP probes, approved drugs, and kinase drugs among LINCS compounds**.

Small molecules tested in different LINCS datasets were compiled, and after removing salts and addends, were submitted to the PubChem web services first for the compound standardization and then for retrieving the PubChem CID identifiers. Unique LSM parent compound IDs were assigned based on the standardized chemical structure representations; a total of 5364 unique compounds were identified across the LINCS assays. Among them, we identified previously known kinase inhibitors, approved drugs, MLP probes, PDB ligands etc. (described in the Materials and Methods). These annotations are illustrated in Figure 1; they are available and can be browsed and queried at the LIFE project website (http://lifekb.org/) and the LIFE search engine (http://life.ccs.miami.edu).

**Figure 2:**
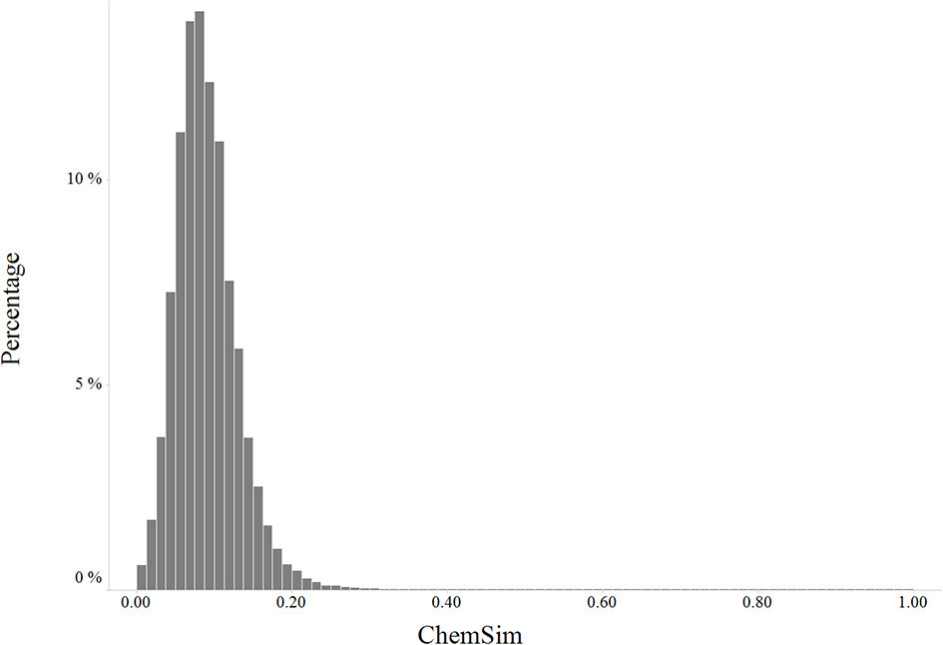
**Distribution of pairwise chemical similarity of LINCS compounds**.

We explored the diversity of compounds in the LINCS chemical space by pairwise Tanimoto similarities based on extended-connectivity fingerprints (Figure 2).

As shown in the similarity histogram (Figure 2), the distribution is skewed toward low similarity suggesting LINCS compounds are fairly diverse (Tanimoto coefficient below 0.4). LINCS compounds were selected by the centers to cover a broad biological space including known drugs, kinase inhibitors and probes from the Molecular Libraries program.

### Overlap of LINCS compounds and cell lines across assays

Cell lines were previously standardized by a joint effort of several LINCS centers (Vempati et al., 2014).

**Figure 3:**
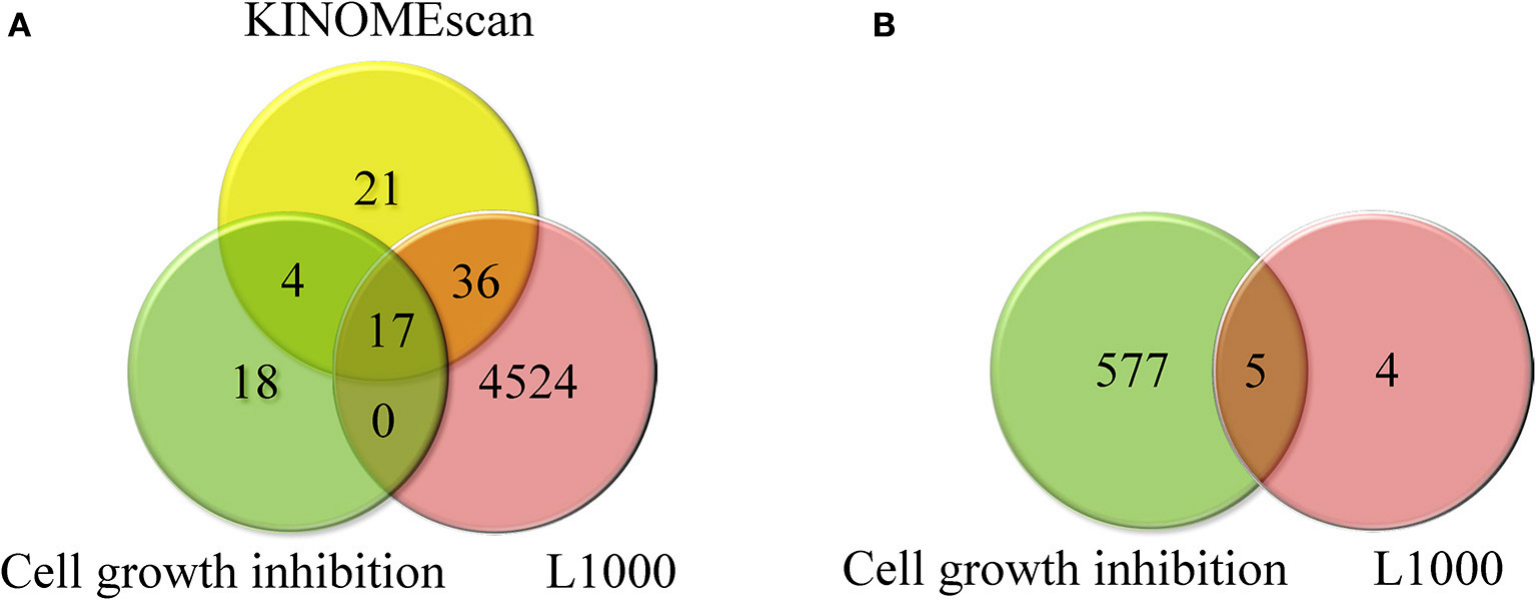
**(A)** Overlap between compounds tested in KINOMEscan, cell growth inhibition, and L1000 assays and **(B)** overlap between cell lines tested in cell growth inhibition and L1000 assays.

Standardized compounds and cell lines were compared across the LINCS Data Generation Centers and selected assays. One hundred and fifty compounds and thirty one cell lines were tested at both centers (HMS and Broad) across different assays. For the assays considered in this study the overlap between tested compounds and cell lines is shown in Figure 3.

From this analysis it becomes obvious that only a small number of compounds were tested in several different assays limiting comprehensive analysis. In order to generate data that would facilitate cross-datasets integration, we built and applied 229 small molecule kinase inhibition models (as described in Materials and Methods) to predict the kinase inhibition profiles for all LINCS compounds. We used these predictions to fill the gaps in the experimental data and to deconvolute the trends between biological responses as described below.

### Integration and analysis of kinase profiling and cell growth inhibition profiling datasets

The integration of Kinome-wide small molecule inhibition profiles and phenotypic responses offer a powerful approach to deconvolute likely mechanisms of action of pharmacologically active compounds. Similar, cell line panels, in particular cancer cell lines, are an established approach to characterize small molecule pharmacologically. Using standardized LINCS KINOMEscan and cell growth inhibition signatures generated for the same compounds enables us to map chemical biology binding profiles to cancer cell viability profiles with the potential to contribute to the identification of key kinases and pathways that are relevant for specific cancer subtypes. To investigate this, we generated all combinations of tested kinases and cell lines and for each combination computed a kinase enrichment score that quantifies how much more likely a compound is to be active if it is an inhibitor of a given kinase over the background probability of inhibiting cell growth (see Materials and Methods). Scores of greater than one indicate that inhibitors of that kinase are more likely to inhibit cell growth, suggesting that the pathways to which these kinases belong may be involved in cell death (desirable outcome for the cancer cell lines). Conversely, enrichment scores of less than minus one indicate that such inhibitors would be less likely to kill the cells.

**Figure 4:**
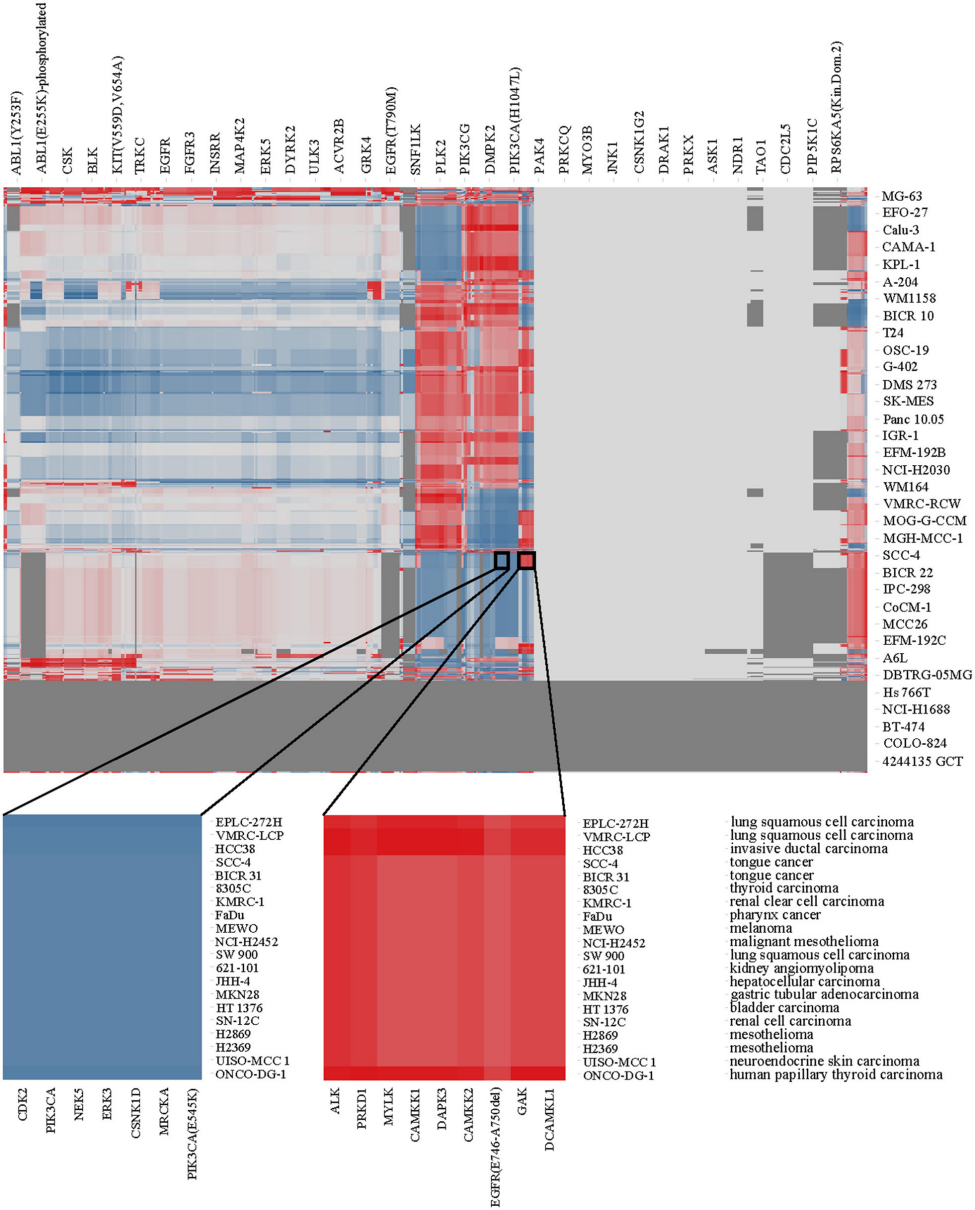
**Heat map of kinase enrichment across cell lines as described in the text**. Two circles focused on the area of high enrichment (red) and derichment (blue), respectively (the gradient color mode range shown from red, for the maximum enrichment score of 3.13, via white for average score of −0.32, to blue color corresponding to minimum derichment score of −5.50). Kinases, cell lines and diseases are annotated.

Using the enrichment scores, we performed hierarchical clustering of kinases and cell lines. The resulting heat map is shown in Figure 4 where red areas represent high kinase enrichment scores, white no enrichment and blue derichment; gray area reflect combinations of kinases and cell lines without overlapping compounds tested in two assays.

### Kinase enrichment and derichment in cancer

Although there is no clear clustering pattern of kinases vs. diseases in Figure 4 (which cannot be expected in a relatively limited dataset and cell line model systems), we can still identify individual kinases that are enriched in certain cell lines. For example, kinases ALK, PRKD1, MYLK, CAMKK1, CAMKK2, DAPK3, EGFR, GAK, DCAMKL1 emerge to be more relevant for the lung squamous cell carcinoma (few cell lines originating from this diseased tissue) while kinases MRCKA, MRCKB, DMPK2, HIPK4, CDK2, CDK8, CDK11, PIK3CA, NEK5, ERK3, and CSNK1D appear to be not affected by compounds causing cell death in the same cell lines. Therefore, after identifying kinases that are enriched in one (or several) disease, one could possibly identify novel drug targets or previously known targets that show activity in a new disease and therefore find a case for drug repurposing. In this way, previously unknown side effects of a compound may be discovered and off-targets can be identified among a subset of enriched kinases.

Our analysis approach illustrates how LINCS data can potentially be leveraged to gain important insight into molecular mechanisms that lead to the cell malignant state, especially in the future with the currently expanding LINCS data. The results shown here should be considered as an illustration for data integration and how they can be interpreted. Even with this limited dataset, we were able to identify several examples of known drugs that would confirm potential conclusions derived from this analysis. For example Lapatinib, an approved drug for breast cancer is very potent in the MCF7 breast cancer cell line by killing 83% of cancerous cells (at 2.5 μM). Its known drug target is EGFR. We also found that this drug inhibits EGFR at 100%, as well as majority of its other modifications/mutations in the KINOMEscan panel.

### Biochemical and phenotypic response signatures are related and interpretable based on chemical similarity

After defining bio-fingerprints to represent cellular signatures generated in the number of LINCS assays (as described in Materials and Methods) we analyzed them to identify correlations and trends between different biological and cellular phenotypic response profiles.

#### Kinome-wide binding activity (KINOMEscan) profiles

**Figure 5:**
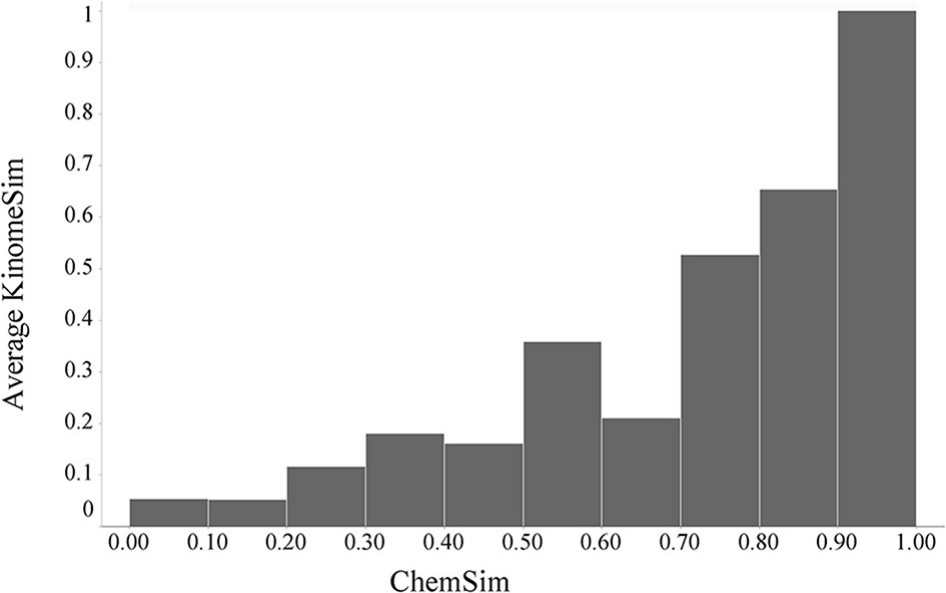
**Global trend of kinase binding profile similarities (KinomeSim) and chemical structure similarities (ChemSim) for 78 compounds, illustrated as average KinomePredSim values by ChemSim ranges**.

We calculated pairwise Tanimoto similarities (KinomeSim) based on the kinase binding (KINOMEscan) profiles for 78 compounds that were tested in that assay (see Materials and Methods). For the same compounds we computed the corresponding pairwise molecular similarities (ChemSim). KinomeSim thus represents the similarity of a compound pair based on their biochemical (kinase) binding profile while ChemSim quantifies the similarity of two compounds based on features of their chemical structures. Chemical structure similarity is an important concept in cheminformatics where it is generally assumed that more structurally similar compounds are more likely to have similar biological activity (similarity property principle) (Martin et al., 2002). Here we apply this concept to a biological profile. Figure 5 illustrates the global relationship between pairwise biological profile and chemical similarities; specifically ChemSim is binned and within each bin the average KinomeSim is calculated and shown as the corresponding bar height. As Figure 5 illustrated, there is a general trend that highly similar compounds also have very similar kinases panel activity (KINOMEscan) profiles. A two-sided Student *t*-test confirmed the statistical significance of this trend. For example using a ChemSim cutoff of 0.8, which can be considered reasonable similar for the fingerprints applied here (see Materials and Methods), the average biological profile similarities of the corresponding KinomeSim distributions are (statistically) significantly different with a *p*-value of 1.9·10–61.

#### Predicted small molecule kinase inhibition profiles

Using predicted kinase inhibition profiles rather than the experimental binding profiles allowed us to investigate a much larger number of compounds. Whereas KINOMEscan profiles were available for 78 compounds, we generated predicted kinase inhibition profiles for all 5364 LINCS standardized compounds as described in Material and Methods. Although we don't expect perfect predictions, we have shown that the predictors are highly accurate (Schurer and Muskal, 2013); we only applied models with sufficient data and very good cross-validation performance. An important characteristic of the kinase classification models is that they are derived from a large corpus of published and patented results comprising many different assay technologies and assay conditions aggregated by unique chemical structures and kinase protein target. It may therefore be the case that such results are in fact more robust in terms of reproducibility as oppose to comparing just two different assay methods or assay conditions, which can sometimes give considerably different outcomes (Haibe-Kains et al., 2013). It was therefore of much interest how the predicted profiles would perform statistically.

**Figure 6:**
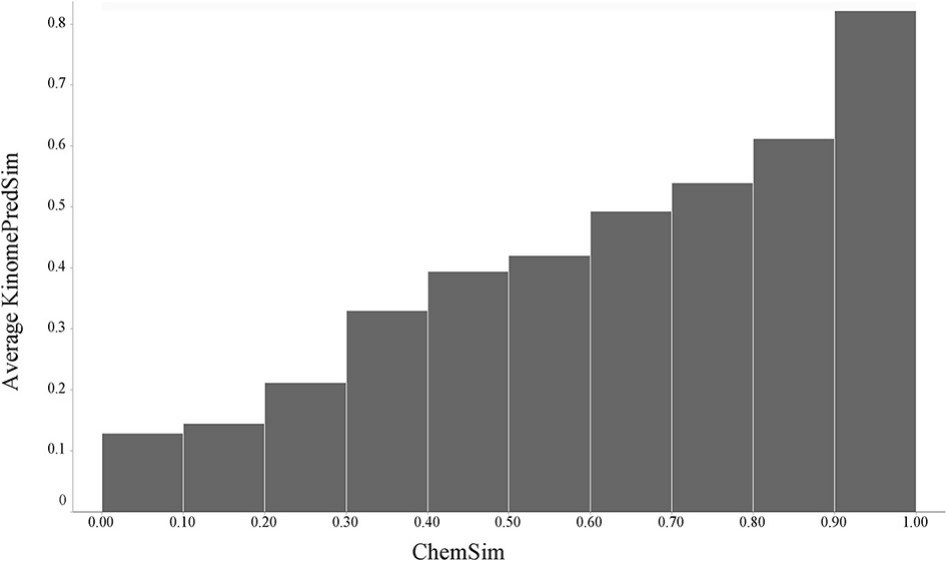
**Global trend of pairwise predicted kinase inhibition profile similarities (KinomePredSim) and chemical structure similarities (ChemSim) for 5364 compounds, illustrated as average KinomePredSim values by ChemSim ranges**.

In the same manner as described above, we compared pairwise similarities based on (predicted) kinase activity profiles (KinomePredSim) and chemical structural features (ChemSim). Figure 6 illustrates the global trend.

As before, structurally similar compounds exhibit similar (in this case predicted) biological response profiles. We corroborated this trend by a *t*-test comparing two distributions of KinomePredSim corresponding to a ChemSim split of 0.8 (reflecting similar and dissimilar compound pairs) and obtained a *p*-value of 1.62·10–79. As expected no such trend is observed when the kinase predictions are randomized.

#### Gene expression (L1000) profiles

**Figure 7:**
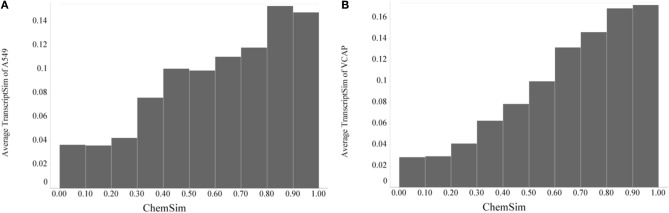
**Global trend of pairwise transcriptional similarity (TranscriptSim) in (A) A549 cells and (B) VCAP cell and chemical structure similarities (ChemSim) for 1027 and 741 compounds per cell line, respectively, illustrated as average TranscriptSim values by ChemSim ranges**.

After demonstrating a robust, perhaps expected trend that the similarity of compounds based on their biochemical activity profiles (KINOMEscan as well as predicted) increases significantly with their chemical similarity, it was of interest to compare chemical similarity to gene expression similarity. To evaluate transcriptional similarity we considered not just one response (active vs. inactive) for each feature (e.g., kinase target), but two responses, overexpressed and underexpressed for each feature (i.e., gene); this was implemented in a binary fingerprint simply by doubling the features as described in Materials and Methods. With that we can again compare pairwise similarities, this time based on the gene expression profiles (TranscriptSim) vs. chemical similarity (ChemSim). We found that a similar global trend holds even in this case, when there are no direct interactions between small molecule perturbagens and the molecular entity underlying the biological profiles, i.e., transcribed gene in this case. Figure 7 illustrates this trend for two dissimilar cell lines, A549 (non-small cell lung carcinoma) and VCAP (prostate carcinoma).

As before we quantified the statistical significance of this trend by the two-tailed *t*-test using a ChemSim cutoff of 0.8 to differentiate similar vs. dissimilar compounds. The *p*-values of the corresponding TranscriptSim distributions are 2.06·10–14 and 9.64·10–14, for the A549 and VCAP cell lines, respectively.

**Figure 8:**
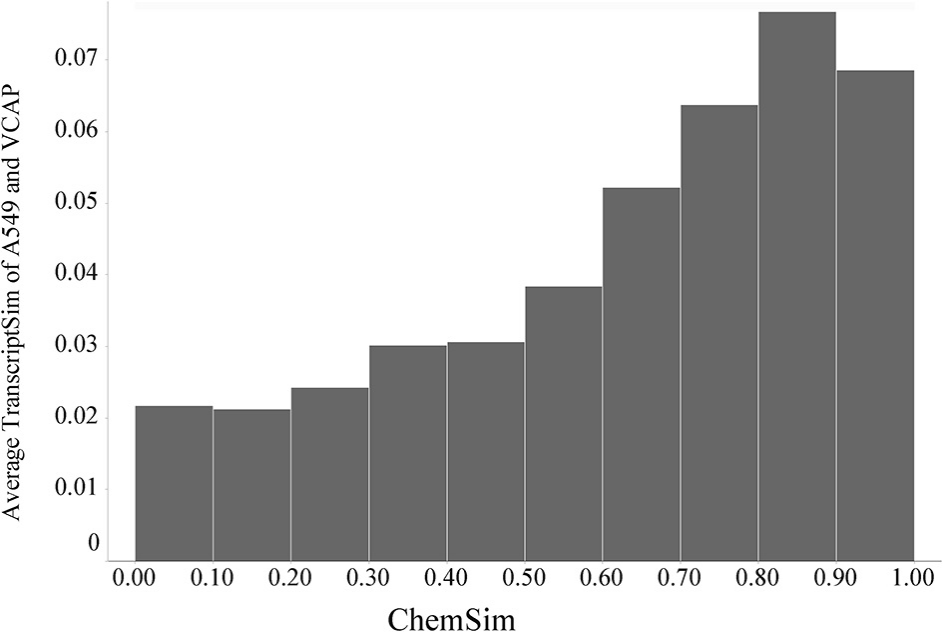
**Global trend of pairwise transcriptional similarity (TranscriptSim) across A549 and VCAP cells and chemical structure similarities (ChemSim) for 1027 and 741 compounds per cell line, respectively, illustrated as average TranscriptSim values by ChemSim ranges**.

In the same way we also compared compound L1000 response profiles across both cell lines. Although there is the general trend of increasing transcriptional similarity with molecular similarity holds, the effect is much smaller (about half the average similarity) compared to the trend on one cell line alone (shown in Figure 8). This is expected, because the cell lines can be expected to have a very different response to the same compounds; in particular that is the case for kinase inhibitors that was evaluated above. The response of kinase inhibitors tested (for example) in A549 and VCAP growth inhibition assays can be explored in our LIFE software (http://life.ccs.miami.edu). A global effect across two very different cell lines is noteworthy and probably related to conserved pathways.

#### Relating small molecule predicted kinase inhibition profiles and gene expression profiles

**Figure 9:**
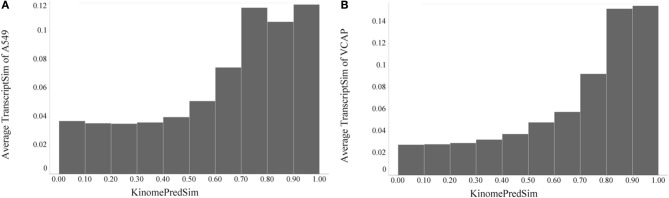
**Global trend of pairwise transcriptional similarity (TranscriptSim) in (A) A549 cells and (B) VCAP cells as a function of predicted kinase profile similarity (KinomePredSim) for 1027 and 741 compounds per cell line, respectively, illustrated as average TranscriptSim values by KinomePredSim ranges**.

After establishing a general global trend of biochemical and transcriptional similarity with compound similarity, it was of interest to compare gene expression (L1000) signatures and kinase inhibition profiles. Because of the limited number of experimental KINOMEscan profiles and encouraged by our results, we compared compound pairwise similarities based on transcriptional response profiles to the predicted kinase inhibition profiles. As shown in Figure 9, compounds that are more similar based on their biochemical kinase profile are also more similar with respect to changes in gene expression. We estimated statistical significance of this trend for the KinomePredSim cutoff of 0.8 (above the cutoff considered similar biochemical kinase profile) with the *p*-values of 1.28·10–21 and 6.70·10–30 for A549 and VCAP, respectively. While it is known that kinases are mechanistically related to downstream gene expression via various signaling pathways and networks, these results suggest some level of global systems-wide stability of gene transcription with respect to modulating the entire human Kinome. We did not incorporate any systems-level information to group kinases (this is described in more detail below), but look only at the global profiles.

**Figure 10:**
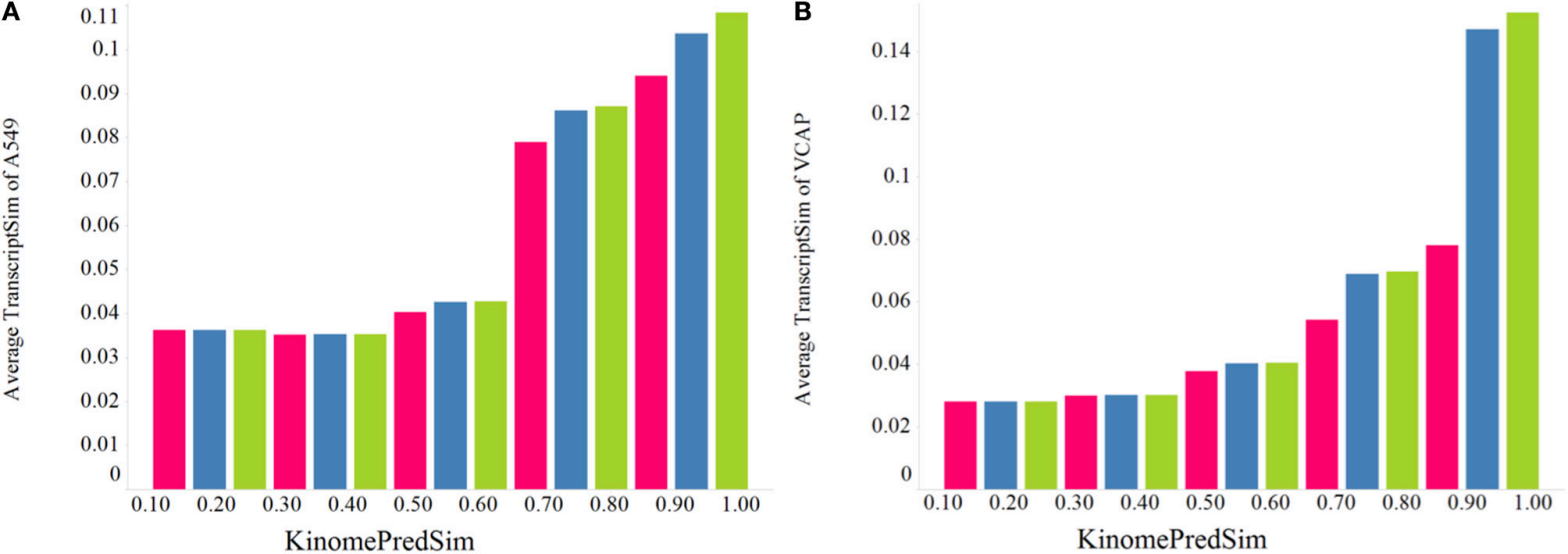
**Effect of the chemical similarity (ChemSim) of compound pairs on the trend of the average TranscriptSim as a function of KinomePredSim in (A) A549 cells and (B) VCAP cells**. ChemSim cutoff applied are: 1.0 (green) including all compound pairs, 0.8 (blue) removing compound pairs more similar than 0.8, and 0.5 (red) leaving only dissimilar compound pairs (ChemSim < 0.5).

Earlier observed trend of increasing transcriptional similarity for more similar chemical purturbagens reasonably could be rationalized based on the assumption that more similar compounds are more likely to bind to similar targets. The kinase profile similarity analyses above confirm that assumption, even at large scale of more than 5000 compounds using predicted kinase profiles. To investigate further the dependencies of chemical similarity, biochemical similarity and transcriptional similarity we analyzed TranscriptSim vs. KinomePredSim for different cutoffs of ChemSim as shown for the two cell lines, A549 and VCAP in Figures 10A,B, respectively. Specifically Figure 10 compares three ChemSim cutoff values, namely 1 (keep all compounds, green), 0.8 (remove compound pairs with similarity higher than that, blue), and 0.5 (leave practically only non-similar compounds, red).

As Figure 10 illustrates, as chemically similar compounds are removed from the analysis, the observed trend between transcriptional similarity and biochemical similarity of compound pairs decreases, but still holds even for only dissimilar compounds (ChemSim cutoff 0.5). This is the case again for two very different cell lines.

**Figure 11:**
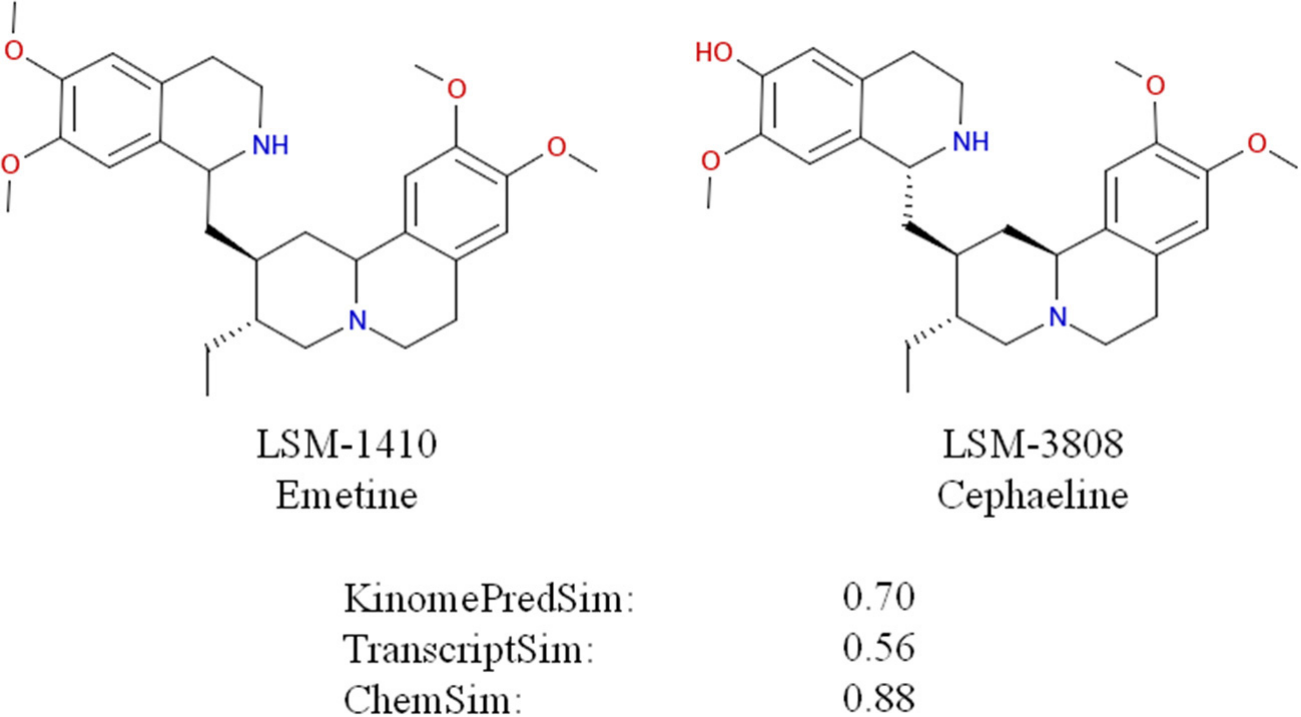
**Similar compounds (ChemSim of 0.88) with high KinomePredSim and TranscriptSim (based on L1000 in A549 cells)**.

To evaluate these trends statistically, we performed Student *t*-tests for the different datasets corresponding to a ChemSim cutoffs of 0.8 (426,331 and 219,163 compound pairs for A549 and VCAP, respectively) and 0.5 (425,452 and 218,648 of compound pairs for A549 and VCAP, respectively). In both cases the dataset was split by a KinomePredSim cutoff of 0.8 (similar and dissimilar based on their predicted kinase inhibition profile) and *p*-values characterizing the difference in mean for the corresponding distributions of transcriptional similarity were calculated. The *p*-values for the ChemSim cutoff of 0.8 are 2.15·10–19 and 1.15·10–28 for A549 and VCAP cell lines, respectively, while for the ChemSim cutoff of 0.5 the *p*-values are 0.004 and 0.014 for A549 and VCAP cells, respectively. These results confirm that the observed trend between the biochemical kinase profile and transcriptional profile similarities is statistically significant even for structurally dissimilar compound pairs. This is noteworthy as a global trend suggesting that transcriptional response signatures may be modeled based on biochemical response profiles alone. With this, it is of course not surprising that this trend is more pronounced with increasing chemical similarity, because—as shown above—chemical similarity would results in higher biochemical similarity. For example, Figure 11 illustrates two highly similar compounds (ChemSim = 0.88) with high KinomePredSim (of 0.70) and TranscriptSim (of 0.56).

**Figure 12:**
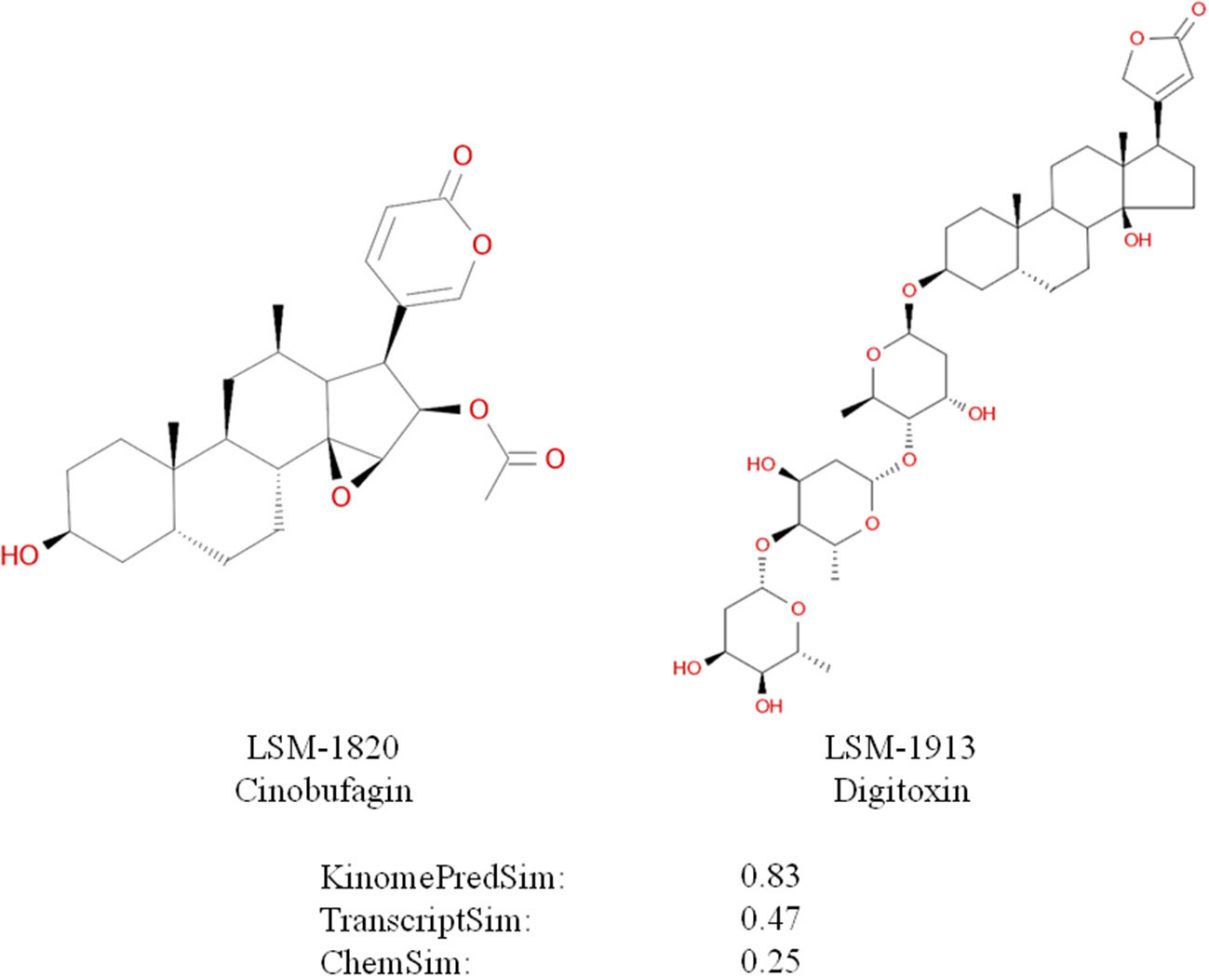
**Dissimilar compounds with high KinomePredSim and high TranscriptSim (based on L1000 in A549 cells)**.

An example of high biochemical similarity and high gene expression similarity for two structurally dissimilar compounds is illustrated in Figure 12; specifically ChemSim = 0.25, KinomePredSim = 0.83, and TranscriptSim = 0.47. Identifying pharmacologically similar, but structurally diverse compounds as demonstrated here using LINCS signatures, is an important approach in drug lead development; for example to overcome undesired physicochemical properties, such as solubility or brain penetration, or for patent reasons.

### Systems-level integration and analysis of LINCS signatures

The above analyses suggested that the transcriptional profiles are correlated (to some extent) to the MoAs of kinase inhibitors as characterized by their kinase inhibition profiles. We therefore anticipated that small molecule perturbagens that affect same pathway would also exhibit similar transcription. To demonstrate that in a specific example, we selected and analyzed the PI3K/AKT/mTOR pathway, which is in the regulation of cell apoptosis and a target of many cancer drug discovery studies. For this example we extracted experimental kinase inhibitor activities from the KKB to identify those compounds that would interact physically with a protein target in the pathway.

In addition we pursued a systematic approach analyzing transcriptional response for all currently available pathways from the NCI database. Here we used the kinase models (described above) to predict LINCS compounds that could affect kinases in the considered pathways.

#### PI3K/AKT/mTOR pathway analysis

For 21 kinases previously identified in the mTOR pathway we identified (using the KKB) 35 active kinase inhibitors among LINCS compounds (see Materials and Methods; see Supplementary Material Dataset 2 for the list of mTOR pathway proteins, 21 mTOR pathway kinases with the inhibition data, and 35 active compounds). For these, pathway-active, compounds we compared L1000 fingerprint similarities. We found that for the two cell lines, the pairwise mTOR pathway inhibitors' L1000 responses are on average more similar then the L1000 responses of all LINCS compounds: for the A549 cell line, the global pairwise L1000 similarity average is 0.035 versus mTOR-pathway compounds' pairwise L1000 similarity average of 0.057; in VCAP cells these number are 0.028 versus 0.043, respectively. The corresponding Student test *p*-values are 1.38·10–28 and 1.1·10–14 for A549 and VCAP, respectively, providing a strong evidence that small molecule perturbagens that interfere with the same pathway (by inhibiting specific kinases in that pathway) result in significantly more similar transcriptional profiles compared to compounds active across different pathways.

#### Systematic pathway analysis

We also performed a more systematic study by using the NCI pathway database. We utilized the kinase inhibition models to predict the most likely pathway-active LINCS compounds in order to cover as much data as possible. We first annotated all kinase targets covered by our models by pathways (using a total of 224 NCI pathways). Once we had the kinase list for each pathway, we identified LINCS compounds that were predicted to be active for kinases in a given pathway, i.e., pathway-active compounds. Pairwise TransciptSim values of these pathway-active compounds were compared to the TranscriptSim numbers of the remaining tested compounds and for each pathway the corresponding *p*-values were calculated. The requirement for the *p*-value calculation for each pathway was the presence of at least three pathway-active compounds, i.e., two similarities between them (necessary for the *t*-test mean distribution calculation). This reduced the number of pathways that could be investigated in formal statistics to 191. For the A549 cell line, 156 of 191 pathways have *p*-value below 0.05, suggesting that the greater transcriptional similarity is not random, while for the VCAP cell line we identified 162 of 191 pathways with *p*-value of less than 0.05. Kinases identified per pathway, as well as pathway-active compounds, can be found in the Supplementary Material Datasets 3 and 4 along with the corresponding *p*-values for cell line A549 and VCAP, respectively.

Even though our approach used a simplified assumption of pathway independence (we analyzed each pathway separately and not as a part of the network), it can be seen that transcriptional expression profiles originating from the same pathway (as defined by the participating kinases) are on average significantly more similar compared to result based on compounds that are not related to the same pathway. This is the case for majority of the pathways. For the pathways where this is not the case, we anticipate that additional information of pathway coexistence and dependence may be needed. However, our results provide strong indication that targeting a particular pathway will most likely lead to a certain transcriptional expression profile. And, importantly, it suggests that we can identify pathway-active compounds based on large-scale published data (KKB) or predict their activity via models based on these datasets.

### Kinase signatures suggest different cell growth inhibition pathways for A549 and VCAP

After illustrating that transcriptional profiles are on average more similar when corresponding to the same cell line then when they are arising from two different cell lines (Figure 8), we were interested to contrast the enrichments of kinases for the two cell lines. We used the enrichment scores (as described in Materials and Methods and depicted in Figure 4) to identify kinases that are relevant for each cell line. Based on the experimental data we found that, for example, kinases PIK3CG, NEK5, ERK3, NEK2, PIK3CA, PRKCE, CSNK2A2, PIM1, PKN2, and CAMK2D are enriched in non-small lung carcinoma A549 cell line while kinases DYRK1B, PCTK1, HIPK1, ICK, CDKL5, DYRK1A, MAK, ERK8, CLK1, and CLK2 are enriched in prostate carcinoma VCAP cell line. Mapping these kinases to pathways suggests that cell toxicity may be mediated by different pathways. For example, for VCAP enriched kinase MAK one pathway was identified from the NCI pathway collection: Co-regulation of Androgen receptor activity. In contrast, for A549 multiple pathways were related to the enriched kinases, but 7 pathways had more than one of these kinases as members: PDGFR-beta signaling pathway, CDC42 signaling events, Atypical NF-kappaB pathway, E-cadherin signaling in the nascent adherens junction, IL3-mediated signaling events, IL5-mediated signaling events, GMCSF-mediated signaling events, IL2-mediated signaling events, Role of Calcineurin-dependent NFAT signaling in lymphocytes, RhoA signaling pathway, IL8- and CXCR1-mediated signaling events, CXCR4-mediated signaling events, Class I PI3K signaling events, Thromboxane A2 receptor signaling pathway. These results illustrate the different (systems-wide) characteristics of the two cell lines and likely underlying mechanisms of action related to their growth inhibition. This is valuable for the development of selective and efficacious drugs based on prioritized and cell line-/disease-specific drug targets.

### Kinase binding and cell viability profiles to guide drug repurposing

In contrast to the example above where there appear to be no common kinase targets, repurposing of known drugs is now a common strategy to quickly identify approved drugs that can be applied to a new disease. Here we show an example of Crizotinib (LSM-1027), approved drug for some non-small cell lung carcinomas. Based on the LINCS KINOMEscan data one can identify kinases that are inhibited by this drug (INSR, AURKB, SRC, IGF1R, ROS1, MAP3K1, TYRO3, EPHB4, AXL, TXK, MET, FGR, FLT3, ALK). Furthermore we can identify the related pathways (NCI pathways described in Material and Methods). Although there are several pathways that may be implicated in multiple diseases, we can also identify specific ones, for example Glypican 1 (NCI Pathway ID 200026), which is associated through kinases SRC and FGR. This pathway is implicated in pancreatic cancer (Aikawa et al., 2008). Therefore by using approved non-small lung carcinoma drug Crizotinib, it may be possible to target SRC or FGR and therefore find its new uses in different cancer types.

## Discussion and conclusions

The LINCS project is a large-scale coordinated effort to generate a comprehensive systems biology reference resource of cellular and molecular response signatures for a wide range of cell lines, primary cells and stem cells, molecular, genetic, and other perturbations. The goals of the program include the generation of a very large multidimensional data matrix and informatics and computational tools to integrate, analyze, and make readily accessible such diverse data as genome-wide transcriptional profiles, biochemical protein binding, large-scale cellular phenotypic response signatures, and also proteomics and metabolomics data. To produce an integrative view of large and diverse datasets like those in the LINCS project, it is important to systematically standardize and annotate all data. Multiple efforts were carried out within our group and the LINCS consortium to define standards specifications and apply them to annotate a variety of perturbing or detected molecular entities cell model systems and other relevant concepts (Vempati et al., 2014). These efforts continue as the project moves into the next phase. Via tools developed in the program, for example the LIFE search engine (http://life.ccs.miami.edu), LINCS data can already be queried by standardized annotations across different sources.

Here we are particularly interested in small molecule perturbations, because of the potential of small molecules to be developed into therapeutic drugs and a general shift from purely target focused toward a systems poly-pharmacology based approach to drug development that could gain great insights from LINCS. To facilitate the cross-comparison of LINCS signatures, we established a fairly automated process for the standardization of small molecule compounds, which simplifies identification of compounds tested across several assays and also facilitates mapping and annotating of compounds using external sources such as DrugBank, the NCBI MLP probe reports, the NPC collection, and the Protein Data Bank (PDB). Unique compound IDs are also required to better coordinate data generation across centers; as illustrated in Figure 3, there are still gaps to be filled in order to achieve a complete data matrix across LINCS assays.

Nevertheless, important insights can be gained by bringing together the current datasets. For example we illustrated the integration of kinase binding profiles (KINOMEscan assay) and cell growth inhibition profiles. We combined these datasets using unique small molecules profiles across and used statistical enrichment to identify kinases that may play a role in the certain cell lines or diseases. The nature of the LINCS data matrix consisting of standardized response profiles enables the prioritization of sets of interesting kinases (signatures) that influence any of the tested cell lines. In that way kinases shared across many cell lines can be identified and such discovery may lead to new target identification or at least novel hypotheses. Also, by discovering common kinases between cell lines related to different diseases may lead to novel starting points for (cancer) drug repurposing.

We demonstrated that the similarity of compounds based on their chemical structure is related to their kinase binding profiles. This could be expected based on the similarity-property dogma, however is still noteworthy at a global level where each profile can represent a characteristic signature, implying that such signatures are related to chemical structures. Looking at the genome-wide transcriptional profiles for a much larger number of tested compounds at the Broad institute (see Materials and Methods), there was a similar trend that relates chemical similarity to global transcriptional similarity. It was more pronounced in the same cell line, but also detectable across cell lines. These chemical similarity trends can be interpreted as a generalization of the classical similarity-property principle, which underlies targeted lead optimization efforts. In particular in the case of transcriptional profiles, which have been related to disease phenotypes and models thereof (Lamb, 2007), these findings appear to support the feasibility of phenotypic lead optimization and utility of phenotypic structure-activity-relationships for drug development.

To link transcriptional responses to the underlying MoA, we compared the transcriptional profiles to the kinase binding profiles. Because of the quite small intersection of compounds for which L1000 and KINOMEscan profiles were available, we developed and applied kinase inhibition classification models based on a very large corpus of published data and applied these to all compounds tested in L1000. In addition to predicting activities for non-tested compounds and extending the current datasets to identify patterns in the data, these computational results can be also used to prioritize compounds for further experimental testing. For example the models could be used to identify a set of diverse compounds that are most likely to efficiently dissect the entire Kinome activity space or to prioritize compounds most likely to interfere in a given biological pathway, or any desirable poly-pharmacology profile to help deconvolute mechanisms of cellular responses.

As expected, the trend we observed for the experimental kinase binding profiles that chemically similar compounds are more likely to have similar kinase inhibition profiles, was also confirmed for the predicted kinase profiles just for all LINCS compounds as the modeling enabled it. We already knew that structurally very similar compounds were also more likely to have similar transcriptional profiles. However, their biochemical kinase similarity appeared related to transcriptional similarity independently from chemical similarity, at least to some extent. This would confirm a mechanistic relationship (by pathways), but more importantly a global response suggests a level of robustness in the cellular responses to chemical perturbation; i.e. small changes in biochemical binding do not have a huge effect on transcriptional response. This may be one reason why most drugs are well tolerated, despite (previously not known) poly-pharmacology and in some cases even alternate indications (drug repurposing). We anticipated that downstream gene expression signatures would be much more closely related by signaling pathways; i.e. compounds inhibiting kinases within a specific pathway should have more similar transcriptional profiles. We tested and confirmed this using actual data for the PI3K/AKT/mTOR pathway and using the kinase inhibition models for a large number of pathways from the NCI database. Although we applied a simplified approach of analyzing individual pathways, we observed that for the majority of pathways the transcriptional expression profiles resulting from small molecules that are active against any kinase in the same pathway are indeed more similar than transcriptional expression profiles of compounds that do not share activity against the same pathway.

Facilitated by common data standards and annotations we were able to integrate diverse biochemical, transcriptional, and phenotypic cell growth inhibition profiles for small molecule drug like molecules. After computing various similarity measures based on the response signatures and chemical information, we illustrated some insightful trends and elucidated the results at the systems-level. Our approach and findings to relate biochemical and transcriptional responses to chemical similarity as well as use of predictive models appear relevant to inform the development of novel poly-pharmacology drugs. We hope that some of the data integration and analysis presented here can inspire others in the research community to leverage LINCS data and the annotations we provided for their own studies and in novel ways.

### Conflict of interest statement

The authors declare that the research was conducted in the absence of any commercial or financial relationships that could be construed as a potential conflict of interest.

## Footnotes

^1^http://lincsproject.org/

^2^http://life.ccs.miami.edu

^3^http://lincscloud.org/

^4^http://www.discoverx.com/technologies-platforms/competitive-binding-technology/kinomescan-technology-platform

^5^http://pubchem.ncbi.nlm.nih.gov/

^6^http://www.drugbank.ca/

^7^http://www.ncbi.nlm.nih.gov/

^8^http://lifekb.org/

^9^http://www.eidogen-sertanty.com/kinasekb.php

^10^http://kinase.com/

^11^http://lincs.hms.harvard.edu/

^12^http://pid.nci.nih.gov/index.shtml

^13^http://www.r-project.org/
