# Supplementary material for: Large-scale integration of small molecule-induced genome-wide transcriptional responses, Kinome-wide binding affinities and cell-growth inhibition profiles reveal global trends characterizing systems-level drug action
Source: Front Genet. 2014 Sep 30;5:342. doi: 10.3389/fgene.2014.00342 (PMC4179751; doi:10.3389/fgene.2014.00342)
Supplement: Supplementary file 1 [file DataSheet1.ZIP › Vidovic - Systems-level LINCS Data Integration - Supplementary Material TOC and Methods.pdf]

Supplementary Material

Large-scale integration of small molecule-induced genome-wide transcriptional responses, Kinome-wide binding affinities and cell-based toxicity profiles reveal global trends characterizing systems-level drug action

Dušica Vidović[1], Amar Koleti[1], Stephan C. Schürer[1,2,]*

[1]Center for Computational Science, University of Miami, Miami, FL, USA
[2]Department of Molecular and Cellular Pharmacology, University of Miami, Miami, FL, USA

*** Correspondence:** Stephan C. Schürer, Center for Computational Science, University of Miami, Gables One Tower 600, 1320 S. Dixie Highway, Miami, FL, 33146, USA.
sschurer@med.miami.edu

1. Supplementary Material

1.1. Description of L1000 Data

1.2. Small Molecule Chemical Structure Standardization

1.3. Bioprofile- and chemical structure-based fingerprints and similarities

1.4. Kinase Enrichment in Cell Growth Inhibition Data

1.5. Dataset1.xlsx (data sheet 1, file)

List of 229 kinases for which the LINCS compounds activity was predicted. Laplacien corrected Naïve Bayesian classification model characteristics such as number of actives compounds, and model validation metrics (ROC score and enrichment factor for 1%; leave-one-out cross-validation).

1.6. Dataset2.xlsx (data sheet 2, file)

Includes list of mTOR pathway proteins, 21 mTOR pathway kinases with the inhibition data, and 35 active compounds.

1.7. Dataset3.xlsx (data sheet 3, file)

Kinases identified per pathway, pathway-active compounds, and the corresponding p-values for the cell line A549.

1.8. Dataset4.xlsx (data sheet 4, file)

Kinases identified per pathway, pathway-active compounds, and the corresponding p-values for the cell line VCAP.

1.1. Description of L1000 Data

The Broad Institute LINCS Center performs large-scale gene expression profiling on the L1000 platform. The L1000 assay is an extension of a previously published method for expression profiling based on Luminex bead technology.[1] The expression profiles are generated for 1,000 landmark transcripts for a range of different cell lines treated with small molecules and genetic perturbagens (gene knockdown or over-expression). The gene expression levels are normalized to the plate controls and profiles (signatures) are generated and reported as Z-scores. The signatures are further characterized based on several quality criteria; here we only used the highest quality level of signatures labeled as "is_gold".

1. Peck D, Crawford ED, Ross KN, Stegmaier K, Golub TR, Lamb J (2006) A method for high-throughput gene expression signature analysis. Genome Biol 7 (7):R61.

1.2. Small Molecule Chemical Structure Standardization

Compound information for small molecule perturbagens was received from the LINCS Data Production centers, HMS and Broad Institute. This information includes sample SMILES, facility compound and sample ID, name, vendor information, etc. To identify unique and common compounds required a rigorous structure standardization pipeline that we implemented for the LINCS program.

SMILES were first converted to SDF (Structure Data File) format by Pipeline Pilot 8.0[1] component 'Molecule from SMILES'. Addends such as salt and solvent fragments were removed based on a previously generated in-house addend library derived from analyzing high throughput screening libraries including the Small Molecule Repository. Structures were then subjected to the PubChem chemical structure standardization procedure using the PubChem[2] Power User Gateway (PUG) service and the resulting standardized SMILES were saved in the text format. In order to identify and assign PubChem CIDs for the retrieved standardized SMILES, we used additional service provided by PubChem PUG. The entire process was automated in a custom protocol using Pipeline Pilot. Unique LINCS small molecule (LSM) IDs were assigned to these standardized (canonical) SMILES, which represent a unique compound (parent) representation. All LINCS compound samples were associated with these LSM IDs. Using this process, a total of 5,364 (as of October, 2013) unique LINCS compounds were obtained.

1. Pipeline Pilot 8.0 (2011). v8.0.1 edn. Accelrys Software Inc., San Diego, CA
2. PubChem. http://pubchem.ncbi.nlm.nih.gov/.

1.3. Bioprofile- and chemical structure-based fingerprints and similarities

To facilitate comparative analysis of LINCS datasets, we defined several bioprofile fingerprints for tested compound. These bioprofile fingerprints were constructed based on categorical outcomes (active / inactive) in the different LINCS profiling assays. The Tanimoto metric was then used as a similarity measure of these profiles. Advantages of this approach include simplicity (binary fingerprints) and computational efficiency (i.e. compute Tanimoto similarities). Chemical similarity

of LINCS compounds was determined based on topological fingerprints derived from the chemical structures also employing the Tanimoto metric. The following fingerprints were defined:

i) KINOMEscan profile: Fingerprint feature represents activity of a considered compound for a given kinase: For each tested compound a profile consisting of 478 kinase inhibition values (corresponding to the KINOMEscan panel) was defined. These profiles were converted into binary fingerprints where the bit value was set to 1 if percent kinase inhibition is greater than 90%, otherwise the bit value was set to 0.

ii) Predicted kinase inhibition profile: Similarly, the fingerprint feature represents predicted activity (active / inactive) of a given compound for a given kinase directly obtained as an output of the model. The binary fingerprint was derived from the Kinome-wide activity profile based on the 229 activity predictions for those kinase models that passed the quality criteria (see above). If a compound was predicted to inhibit a kinase, a corresponding bit value was set to 1, and if not the bit value was set to 0.

iii) Transcriptional expression (L1000) profile: L1000 data was treated in a slightly different way to encode information on over- and under-expressed genes. Two 1000-bit fingerprints, corresponding to over- and under-expressed genes, respectively were derived from the original L1000 (Z-score) signatures and combined to one 2000-bit fingerprint. The genes were defined as over-expressed if the Z-score was greater than 2 and under-expressed if the Z-score was less than -2; the values for the bits corresponding to over- and under-expressed genes were set accordingly, 1 if the gene was over- or under-expressed, respectively, and 0 if not.

iv) Small molecule topological fingerprints: To characterize small molecules we used extended-connectivity fingerprints of length 4 (ECFP4)[25].

We use the following terms to refer to the Tanimoto similarities of two compounds based on the above fingerprints:

KINOMEscan profile: KinomeSim

Predicted kinase inhibition profile: KinomePredSim

Transcriptional expression (L1000) profile: TranscriptSim

Small molecule topological fingerprint: ChemSim.

1.4. Kinase Enrichment in Cell Growth Inhibition Data

For each kinase - cell line combination, we consider activities of these 21 common compounds in the two assays. For the purposes of this study we consider a compound active in the KINOMEscan assay if the inhibition is greater than 90% and active in the cell growth inhibition assay if the cell viability is less than 20% (corresponding to growth inhibition of greater than 80%), which corresponds to approximately two standard deviations below the mean, for at least one tested concentration. For

each kinase k we calculated an enrichment score to reflect how much more likely it is to find activity in the cell growth inhibition assay among compounds that inhibit kinase k over the background probability of a compound inhibiting cell growth. To account for the different frequencies of compounds active against any single kinase (and not to overstate the effect of a very few actives), we add a Laplacien correction as shown in equation (I); the Laplacien correction stabilizes the enrichment score as the number of kinase actives approaches zero, the enrichment score approaches one (no enrichment).

$$E_{corrected} = \frac{\dfrac{S_k + 1}{T_k + \dfrac{T}{S}}}{\dfrac{S}{T}} \quad \text{(I)}$$

where:

T - total number of tested compounds

Tk - number of compounds active in the KINOMEscan assay against kinase k.

S - number of compounds active in the cell growth inhibition assay (for the given cell line)

Sk - number of compounds active in both the KINOMEscan assay or kinase k and the growth inhibition assay (for the given cell line)

To make this score more intuitive and facilitate visualization we apply the transformation in equation (II) if the kinase enrichment score is less than one, i.e. if there is derichment:

$$E_{corrected} = -\frac{1}{E_{corrected}} \quad \text{(II)}$$
